# Young Spouses' Experiences of Having a Partner With Heart Disease and Adolescents Living at Home

**DOI:** 10.1111/hex.14129

**Published:** 2024-07-05

**Authors:** Matilda Holmbom, Frida Andréasson, Hanna Grundström, Camilla Bernild, Nina Fålun, Tone Merete Norekvål, Selina Kikkenborg Berg, Anna Strömberg

**Affiliations:** ^1^ Department of Health, Medicine and Caring Sciences Linköping University Linköping Sweden; ^2^ Department of Social Work Linnaeus University Kalmar Sweden; ^3^ Department of Obstetrics and Gynecology Norrkoping Sweden; ^4^ Department of Biomedical and Clinical Sciences Linköping University Linköping Sweden; ^5^ The Heart Center, Rigshospitalet Copenhagen University Hospital Copenhagen Denmark; ^6^ Haukeland University Hospital Bergen Norway; ^7^ Department of Clinical Science University of Bergen Bergen Norway; ^8^ Department of Cardiology Linköping University Linköping Sweden

**Keywords:** adolescents, heart disease, informal caregivers, parenting, qualitative research, spouses, thematic analysis

## Abstract

**Aim:**

To describe the life situation of spouses having a partner with heart disease and adolescents living at home.

**Design:**

Qualitative inductive design.

**Method:**

Participants (*n* = 22) were included from three Scandinavian countries. Semi‐structured interviews were analysed using thematic analysis with an inductive and latent approach.

**Results:**

Three themes were derived. ‘Being in spousal and parental role transition’ described how daily life had been affected and parental responsibilities had been doubled due to their partner's heart disease. ‘Living with unpredictability and insecurity’ included how the unpredictable illness trajectory caused worries and affected the well‐being of the family. ‘Managing a challenging life situation’ highlights how spouses coped with their partners' heart disease and adapted to a new life situation.

**Conclusion:**

Young spouses' life situation was greatly affected by their partner's heart disease, resulting in increased responsibilities and double parenthood. Having a positive attitude and mindset towards life was used as a strategy to cope with the changed life situation and find a new way of life.

**Implications for the Profession and/or Patient Care:**

All family members are affected by heart disease. Spouses needed additional professional support and guidance on how to involve the children when a parent is ill.

**Impacts:**

- This study highlights how young spouses, with adolescents living at home, experience their life situation.
- The life situation is unpredictable due to the partner's heart disease, as they must handle both caring for their partner and taking on double parenthood.
- Research involving family members can improve person‐ and family‐centred care and treatment outcomes in health care and society.

**Reporting Method:**

COREQ checklist was used preparing the manuscript.

**Patient or Public Contribution:**

Data collection included interviews with spouse.

**What Does This Paper Contribute to the Wider Global Clinical Community?:**

By highlighting the spouses changed life situation due to heart disease and the importance of including them in health care.

## 1 Introduction

The chronic nature of heart disease requires the person being ill to make adjustments to many areas of life, often necessitating support from an informal caregiver, such as a spouse, child or friend who provides unpaid care [1]. Informal caregivers contribute to the recovery process by promoting self‐management, empowering care recipients and increasing adherence to therapy [2, 3]. In the cardiac context, caregiving activities can include monitoring blood pressure, participating in exercise and coordinating and attending medical appointments [2]. Including the family in care enhances the potential for health restoration and improved quality of life [4, 5, 6]. Furthermore, involving informal caregivers can strengthen their ability to cope with the thoughts and worries associated with heart disease [3].

Being an informal caregiver can bring positive aspects, including a sense of being needed, deepening the relationship with the person with heart disease and dedicating time together [7]. However, many informal caregivers experience feelings of insecurity in their role and a lack of preparedness to provide the expected or necessary support and care [4]. Consequently, they require additional support, information and guidance from the healthcare system [3, 4]. Insufficient support may expose informal caregivers to negative consequences that can result in their own ill health [4, 8].

When the informal caregiver is a spouse, the changes that occur within the couple's relationship can create an imbalance within the family. The dynamics of the relationship are influenced by the fact that one person assumes the role of providing support while the other person relies on that support. Caregiving can become a defining role for individuals, and many spouses who care for someone tend to primarily associate themselves as informal caregivers [9]. When the spouse is also a parent, the caregiving role becomes even more complex. Balancing the demands of caregiving with the responsibilities of parenthood introduces additional challenges. This dual role may lead to significant stressors and strain, as caregivers strive to provide emotional and physical support to both their partner and children [10].

The role of spouses as informal caregivers undergoes changes over time due to the unpredictable trajectory of the illness. The duration of caregiving can vary, ranging from a limited period to a lifelong commitment in the case of chronic illness [11]. Being an informal caregiver entails prioritising the needs of the partner over one's own well‐being [9], often involving an intense focus on the partner´s illness. Spouses of partners with heart disease often experience physical, emotional and psychological burdens in their caregiving role, such as anxiety, stress and feelings of guilt or inadequacy [12]. It is common for spouses to experience limitations in their daily lives, as an increased amount of time may be required to provide support for the partner with heart disease [13, 14]. The unpredictable nature of heart disease symptoms and concerns about the deteriorating condition of the partner often result in spouses perceiving that their activities primarily revolve around managing the escalation of symptoms [3].

Having a partner diagnosed with a chronic disease at a younger age is more unexpected than the disease occurring later in life. Neither family members nor the affected person is prepared for the physical and lifestyle changes that come with the illness [15]. The situation may be more difficult to manage for informal caregivers while at the same time working, engaging in caregiving, taking care of the family and having the main responsibility of the household [16]. Given the impact of a cancer diagnosis on the family [17, 18], we have identified a notable knowledge gap in the literature regarding how heart disease specifically affects family life. It is important to explore the unique challenges encountered by spouses who navigate the complexities of supporting a partner with heart disease while also fulfilling the duties and obligations of parenthood.

## 2 Aim

To describe the life situation of spouses having a partner with heart disease and adolescents living at home.

## 3 Methods

### 3.1 Design and Setting

The study has a qualitative inductive design, conducted in three Scandinavian countries.

### 3.2 Theoretical Framework

We have chosen to describe thematic analysis within a constructionist paradigm. From this standpoint, individuals are actively engaged in constructing their own realities. The authenticity of knowledge in the social context emerges through interactions and shared experiences. This dynamic process involves the exchange and co‐construction of ideas within a community, contributing to a collective understanding that shapes individuals' operating context [19]. The thematic analysis enables us to comprehend the social, cultural and structural contexts that influence individual experiences for a deeper understanding of meaning [20, 21].

### 3.3 Study Participants and Inclusion Criteria

There were 22 participants included from Sweden (*N* = 8), Denmark (*N* = 4) and Norway (*N* = 10). Participants were recruited via the patients who were cared for at cardiology units at three university hospitals, meeting inclusion criteria: spouses of a parent with one of the most prevalent heart diseases (ischaemic heart disease, heart failure, and arrhythmia) between 6 months and 5 years since diagnosis, and with adolescents between 13 and 19 years old.

The participants consisted of 16 women and six men, aged varied between 45 and 56 years with a median of 47 years old. Age was not documented for all. They had a range of one to three children in the family with a median of two children. The median age of the children was 16 years. The youngest member in the family was 4 years old and the oldest was 42 years. Eighteen spouses were married to the person being ill. Two were romantic partners living together. Two were divorced from the person being ill and lived alone or with a new partner. Two additional families were approached: the first declined participation out of respect for an adolescent's discomfort, while the second faced language challenges.

### 3.4 Data Collection

Nurses screened patient records or asked hospitalised patients to verify inclusion criteria for adolescents at home. Then, eligible patients were provided with both oral and written information about the study. After 1 week they were contacted by the research team and asked if the family wanted to participate. Written informed consent from all the participants in the families was obtained. They were also made aware of their right to withdraw from the study at any time, without the need for an explanation.

Interviews were conducted between April 2019 and June 2022. Three different researchers conducted the interviews in their own country, in their native language. Before the Covid 19 pandemic spouses were interviewed in a location of their choice, either in their home or hospital areas. During the Covid 19 pandemic spouses were interviewed via Zoom or telephone. Of the interviews, four were conducted face‐to‐face, six via Zoom and 12 via telephone. A semi‐structured interview guide was used with questions based on different themes regarding the spouse's life situation; heart disease in the family, children and parenthood, health and wellbeing, the disease's impact on work and social life and the need for support. In the current study, only the data from interviews with the spouses were used. A similar interview guide was used in all three countries. The interviews had a median time of 35 min, an average of 43 min and lasted between 22 and 97 min. All interviews were recorded and transcribed. An administrator transcribed the interviews, and the transcripts were checked by the interviewer afterwards. To ensure confidentiality, the data were securely stored in locked electronic files on the researcher's server. All the quotes were translated from their native language into English for presentation. The research team discussed the translation to reach a consensus before the presentation.

### 3.5 Data Analysis

Data were analysed using thematic analysis with an inductive and latent approach [20, 22]. NVivo and Microsoft Word® were used to organise the data. The analysis process followed the phases of thematic analysis, but as the analysis process is iterative, it was moving back and forward throughout the phases. The transcribed data were read through several times to get an overall understanding of the content. During this initial phase, preliminary ideas were noted down and continued throughout the analysis process. Coding was then conducted to condense the data and achieve a conceptual interpretation of the transcriptions. We continually revisited earlier phases of the coding for validation, with each repetition building upon the insights gained from the previous one. It allows for refinement, adjustment and improvement; this leads to a more thorough exploration and development of robust findings.

In the subsequent phase, searching for emerging themes was initiated by gathering relevant data associated with each potential theme. The themes were subsequently organised into broader themes by identifying recurring patterns and assessing their interrelationships. This iterative analysis process involved the refinement and naming of themes to generate clear descriptions. Ultimately, themes and sub‐themes were developed [20]. The first author led the analysis process, while the whole research team from all three countries was engaged in discussions to ensure consensus at each step.

To protect data integrity, no data were transferred between countries, the research team convened face‐to‐face on multiple occasions, collaborating closely during the initial stages of the analysis process. The initial coding phase was conducted collectively. Subsequently, each team member responsible for conducting interviews undertook the coding of data from their respective countries. During intervals between meetings, researchers from each country independently worked on their data, reconvening as a team to reach an agreement. Quotes were exchanged between teams to facilitate consensus. Given the similar cultures and languages of the three Scandinavian countries, communication in their native language was comprehensible among team members.

### 3.6 Ethical Considerations

This study conforms with the principles in the Declaration of Helsinki [23]. The study was initiated after receiving ethical approval from the ethical authorities in the respective countries.

### 3.7 Rigour and Reflexivity

To establish and maintain rigour, the multidisciplinary research team's diverse expertise and backgrounds were leveraged. The first author is a R.N., midwife, lecturer and PhD student. The second author is a sociologist, senior lecturer in social work and has a PhD in Health Science. The third author is a R.N., midwife and associate professor. The fourth author is a R.N. and PhD. The fifth author is a R.N. in intensive care and MSc. The sixth author is a R.N. and a professor in cardiovascular nursing. The seventh author is a professor in cardiac nursing. The last author is a professor in nursing and a heart failure nurse specialist. The multidisciplinary research team possessed general knowledge about heart disease, but it is important to emphasise that their preconceptions were more general and not particularly adapted to the specific target group of the study. Despite having insights about heart disease at a general level, the authors consciously went into the study with an open mind. During analysis and reporting, the focus was on looking at the data from different perspectives to increase reflexivity. In summary, rigour was established and maintained through the multidisciplinary composition of the research team, their open‐minded approach to the study and the deliberate effort to examine the data from diverse perspectives to increase reflexivity.

## 4 Findings

Three themes with sub‐themes were derived from the analysis process (Table 1).

**Table 1:** Themes and sub‐themes.

**Themes**	Sub‐themes
Being in spousal and parental role transition	1. Increasing responsibilities in everyday life 2. Burden of double parenting
Living with unpredictability and insecurity	1. Constant worries 2. Disappointments and frustration 3. Limited possibilities for planning ahead
Managing a challenging life situation	1. Asking for help is both difficult and lifesaving 2. Strategies for protecting the children 3. Making the most of life

### 4.1 Being in Spousal and Parental Role Transition

The first theme describes how spouses have been affected by their partners' heart disease and the impact on everyday life and parenthood. Spouses described a transition in their role as a spouse while taking on more responsibility.

#### 4.1.1 Increasing Responsibilities in Everyday Life

Since the partner was diagnosed with heart disease, spouses assumed greater responsibility for the family and the logistics of everyday life, including household chores, managing various contacts and organising activities. This responsibility could result in feeling obligated to stay home and fulfil family duties.

It was just something to deal with … it ended up mostly on me, and it has continued to do so.

(Swedish wife no. 4)

They perceived a sense of expectations placed on them, and that they must adapt to the new life situation to meet the family´s needs, even if they were not sufficiently prepared.

Spouses expressed worries for their partners, about their worsening condition if they were required to undertake tasks. Therefore, they demand less, and make more effort themselves, to relieve pressure and show consideration towards their partners. Some spouses took on the role of a caregiver for their partners, which involved practical support in areas like medication management and facilitating communication with healthcare professionals.

I became like a nurse at home. I was on sick leave after he got ill, just to be able to take care of him.

(Norwegian wife no. 1)

This caregiving role could be burdensome for certain spouses. However, some spouses viewed the caregiving role as a natural and inherent part of their relationship, embracing their responsibilities willingly.

The working situation of some of the spouses had been affected, leading them to make efforts to alleviate the situation. Some considered giving up paid work and assuming the role of a full‐time caregiver. Additionally, spouses assumed the responsibility of conveying information about their partners' condition to their family and friends.

Yes, but informing everyone, everyone wants to know things, so that was probably the heaviest part, that everyone wondered so much … They asked again and were persistent; ‘why is it so?’, so there were a lot of information and talking to them.

(Swedish wife no. 2)

This responsibility was perceived as burdensome, as they constantly had to inform everyone else about their partner's condition.

#### 4.1.2 Burden of Double Parenting

Some spouses expressed the need to compensate for the absence of the other parent yet found it challenging to find time for rest themselves. Some felt constrained from engaging in activities they had previously enjoyed, as they were solely responsible for taking care of the children.

You're alone and feel helpless, and I mean if you only had someone … and then you feel like you're the lonely mother in the whole world and you have to manage everything on your own … then it's very lonely.

(Swedish wife no. 3)

The altered experience of parenthood was characterised by feelings of loneliness and vulnerability due to the absence of the other parent. Consequently, the burden was intensified, as they had to fulfil the roles of both parents.

He is not a full‐time parent, he can´t be. It's not that he doesn't want to, he's very active with the kids, but he can't do all that. He can't take them out like other parents, but he can do other things. He loves to go to flea markets with the youngest daughter. They have their thing that are unique to them, but of course it is a different life and the children have to live with it.

(Danish wife no. 2)

The presence of children at home was seen as a potential source of relief for some of the spouses. However, it also brought an additional responsibility, as they felt accountable for supporting and caring for their children, rather than solely focusing on themselves. Managing the emotional needs of adolescents and addressing their concerns about a parent's illness was perceived as a unique dimension accompanied by concurrent caregiving responsibilities.

Well, it's a completely different situation if you have children at home as I see it, when you're old and retired you don't have the same things to think about like getting a job and so on, so that would be a pretty big difference, I imagine.

(Swedish husband no. 4)

Parenting was acknowledged as a demanding task even without the presence of illness, but now the family had to navigate the complexities of managing a serious illness alongside their parental responsibilities.

### 4.2 Living With Unpredictability and Insecurity

The second theme sheds light on the apprehensions and anxieties of spouses regarding the unpredictable illness and its effects on their own and the family's well‐being.

#### 4.2.1 Constant Worries

Spouses expressed worries about various aspects related to their partner's heart disease, such as the progression of the illness, potential complications, the effectiveness of treatment, managing daily tasks, financial implications and the overall impact on the family's well‐being. The life situation was perceived as uncertain, characterised by a sense of unpredictability and a lack of readily available answers to their inquiries.

Well, it has affected me a lot because I haven't been a person who was worried. I haven't had any catastrophic thoughts or anything like that, but I have become more worried. I actually sought healthcare myself to make sure there was nothing wrong with me, but they said it was psychological stress.

(Swedish wife no. 5)

Spouses had to adapt to living with this uncertainty, which could result in a reshaping of their sense of self‐perception. Some of the spouses believed that stress could exacerbate the disease and vigilantly observed their partner for any changes. Due to the COVID‐19 pandemic, spouses and families were prohibited from accompanying their partners to healthcare visits. Consequently, they experienced a sense of being excluded from their partners' care and were deprived of important information.

#### 4.2.2 Disappointments and Frustration

Some spouses observed their partners displaying signs of fatigue and reduced energy levels. They also noticed heightened irritability and decreased stress tolerance, resulting in a strained atmosphere at home.

I simply can't stand it, so I actually go to work to get away, because I can't sit here, it drives me crazy.

(Danish husband no. 1)

Spouses also experienced that their partner was not as mentally present as before, which caused frustration and disappointment.

I notice that he does not really have the energy to be as mentally present as he has been before. He escapes a bit by looking at his phone.

(Swedish wife no. 1)

Some spouses conveyed frustration and disappointment regarding their partners' reduced engagement in activities and limited ability to take care of their health. They perceived the occurrence of heart disease at a younger age as more unexpected and believed it had a significant negative impact on the family.

He has a lot in common with people who are like 65+, they eat the same medications and have the same problems. It´s the same when he has been hospitalized, there is no one his age.

(Swedish wife no. 6)

#### 4.2.3 Limited Possibilities for Planning Ahead

Spouses faced limitations in planning activities due to the unpredictable nature of the disease. They hesitated to make long‐term plans, fearing the need for cancellations or withdrawals.

We take one day at a time, that's often how we do it, because it's hard to plan the emotions five days in advance.

(Danish wife no. 3)

This avoidance was driven by concerns about causing stress or inconvenience to others. Some spouses also faced constraints in engaging in certain activities, such as refraining from longer trips due to potential medical emergencies.

Now it's not safe for my daughter to go to the amusement park with dad anymore, you can get heart attacks from it, right? It's a shame, because it's one of the things that dad and daughter do together.

(Danish wife no. 4)

This limitation caused feelings of guilt towards their families and a sense of reduced capability to participate fully in activities.

During the Covid‐19 pandemic, some spouses experienced the restrictions as a positive excuse to avoid socialising, finding relief in not having to invite others over. They continued with limited social interactions even after the pandemic as everyone had grown accustomed to it.

### 4.3 Managing a Challenging Life Situation

The last theme highlights how spouses have coped with their partners' heart disease, supported their children and adjusted to a new way of life. They shared various strategies to overcome the challenges posed by heart disease.

#### 4.3.1 Asking for Help is Both Difficult and Lifesaving

Spouses found it difficult to ask for help initially but realised its importance considering their partners' heart disease. They appreciated the support received from their social network, including help with household chores, childcare and food delivery. However, some expressed a lack of professional support from the healthcare system, especially after leaving the hospital, and expressed frustration in reaching out to a doctor or a nurse.

When my husband woke up, I was no longer getting the daily information from the intensive care. He was getting lots of information, but he couldn't absorb what he was being told—so I was only getting bits and pieces. I was very uncertain about what it would be like to get him home, what would it do to us?

(Norwegian wife no. 2)

Some spouses experienced that healthcare professionals often neglected to address the needs of the family, particularly the presence of children.

We have never been informed as a family. There was never any mentioning of the children and what information they received. I think we just got a brochure.

(Norwegian wife no. 3)

They needed guidance on discussing the disease with their children and stressed the importance of children receiving information from healthcare professionals as well, not just from their parents. There was a demand for a clearer and more organised care structure, as it was perceived as complex and unclear. Some spouses experienced a lack of continuity without consistent contact with a single doctor.

The most important thing is that you don't have to sit alone at home with your questions, that you have someone to ask. I can imagine that there are more questions the younger you are somehow. Older people take it, it's like, I don't know, it's more, it comes in the package of getting old with a different acceptance.

(Swedish wife no. 3).

Since heart disease often affects older people, some spouses suggested a need for more information and greater support when heart disease affects younger people.

The children's school provided a much‐needed respite from the disease for the spouses, allowing the children to experience a sense of normalcy.

It was very good to have a teacher who cared about my son. First it was text messages, and then he has been calling. So having an extra adult who took some responsibility, it was very good. And it was very good for my son.

(Norwegian wife no. 2)

The involvement of others, like school counsellors, in caring for and talking to the children was also greatly appreciated.

#### 4.3.2 Strategies for Protecting the Children

Spouses used different strategies to manage the impact of heart disease on their families. Some openly discussed the situation, finding support in sharing their feelings and normalising life with heart disease. Others chose to minimise the disease's significance, protecting their families from excessive anxiety by providing limited and simple information to the children.

We have talked very little about their dad´s heart disease. I can't say what it has done to them, but I have tried to keep it down a bit, because I don't want to create a state of panic, that they should go around and worry about him.

(Norwegian wife no. 4)

Spouses shouldered the responsibility of minimising the negative impact of the other parent's heart disease on their children. This increased their stress and burden as they had to manage their own anxieties while supporting their dependent children. Concerns arose about making sacrifices during adolescence due to the parent's condition, prompting spouses to compensate and alleviate any potential negative effects.

It's hardest to see her (the youngest daughter) take a back seat. The oldest one lives with her dad every other week. She has a different set of reins.

(Swedish wife no. 3)

The balance between involving the children and shielding them from distress was a constant challenge for the spouses.

#### 4.3.3 Making the Most of Life

Some spouses adopted a positive attitude and mindset towards life, enabling them to cope with the challenges brought about by the presence of heart disease in their families. While they did not choose the new life situation, they emphasised their ability to choose how to approach it. They believed that there was always something to appreciate in life, regardless of the circumstances.

One thing that has affected is that you are very grateful for small things. I could have been a widow today … especially the first year was very much like: oh, how wonderful that he is still with us.

(Swedish wife no. 5)

Some spouses described a strengthened relationship with their partner. The experience served as a reminder of life's impermanence, prompting them to cherish each other and not take their presence for granted.

It is important to make the most of everyday life. I think we used to do that too, but maybe even more so now. We've always had that kind of family cohesion, it was a strength we had from before. We have seen how important it is in a situation like this.

(Norwegian wife no. 5)

They had learned to cherish and make the most of each moment, finding gratitude in everyday experiences.

## 5 Discussion

The aim of the study was to describe the life situation of spouses with a partner with heart disease and adolescents living at home. Spouses faced significant challenges and life changes. They assume heightened responsibilities, experience vulnerability and loneliness in their parental role, and lack professional support. However, they developed coping strategies and adapted to their partners' illnesses and new circumstances.

Spouses faced significant challenges in their life situations due to their partners' heart disease. They found themselves taking on increased practical responsibilities and could no longer rely on their partner in the same way as before. The findings align with previous research that highlights the impact of heart disease on informal caregivers, who often assume responsibility for managing daily life [3, 5, 15]. The findings of our study are consistent with previous research on the life situation of spouses caring for individuals with conditions, such as dementia [24, 25, 26] or mental illness [27]. However, heart disease is characterised by an unpredictability that adds an additional layer of stress and anxiety for both patients and caregivers [28]. Providing care to someone can have an impact on the person´s sense of self‐perception, creating a conflict between personal independence and caregiving responsibilities [9]. Andréasson emphasises the expectations placed on spouses as responsible caregivers and the challenges that come with this role. Informal caregivers may perceive themselves as being overshadowed by the person they care for [29]. In our study, some spouses served as caregivers for their partners, which can further affect the balance of the relationship. They may find themselves having to learn and perform nursing tasks, which can be overwhelming and leave them feeling as if they are neglecting their own well‐being. This caregiving role may not be a choice but a necessary duty that must be fulfilled [30]. Our findings indicate that spouses have different perspectives on caregiving, with some considering it a natural part of their relationship, while others find it more burdensome.

Younger spouses, when caring for partners with chronic diseases, face the challenge of balancing caregiving with parenting. Our findings show that spouses often adopt a protective buffering strategy, assuming the role of managing their children's emotions and compensating for the parent being ill. The spouses strive to alleviate any negative consequences for their children.

Despite the challenges, having children at home could also bring comfort. Our findings align with previous research [31], which emphasise the impact of cancer on parenting and family dynamics. Similarly, our study reveals that heart disease also affects parenting and alters family roles.

Women are the primary informal caregivers, especially among working‐age spouses [32]. They often provide higher‐intensity care and perceive it as more demanding than men [33]. This gender distribution is consistent in the context of heart disease caregiving [30]. Women also assume the primary responsibility for caring for children [9]. Women's health is more negatively affected by caregiving, particularly if they have paid employment, leading to less personal time and an increased risk of isolation [33, 34]. Combining caregiving with full‐time employment can have larger negative mental health effects [35]. Living together with the care recipient is most common among caregivers and can increase the pressure [32, 34]. We strived to have a sample that reflected the diversity of relationships in society. Even though some couples were divorced, the majority lived together or were married. Earlier research shows that negative relationships worsen caregiver experiences, while positive ones offer rewards [36]. Unsatisfactory marital relationships and partner disagreements are linked to burnout, emphasising the need for cooperation [1]. In our study, the divorced parents did not experience disagreements and were able to communicate and collaborate effectively despite not living in the same household.

Spouses caring for partners with heart disease often experience constant worry for their partner's well‐being, which is consistent with previous research [30, 34]. While some spouses in the study reported gradually learning to live with the worry, it remained a constant concern due to the unpredictable nature of heart disease progression. Unlike some chronic conditions with a more predictable trajectory, heart diseases often lead to sudden acute events, which may heighten worry.

Spouses also expressed a sense of being overlooked by healthcare professionals and a lack of preparation and knowledge in their caregiving role. This aligns with previous research highlighting the desire for greater involvement and support from healthcare professionals among informal caregivers [5, 37]. In our study, spouses felt marginalised in healthcare settings, with all the focus on the person being ill and little consideration for the family. They were not even asked about the involvement of their children. Similarly, Inhestern [38] found that spouses desired information and support on how to navigate the impact of the illness on their children. Including family members in the care process is crucial for effective care [39], emphasising the importance of healthcare professionals proactively addressing the needs of the whole family and providing preventive support.

Despite the challenges, spouses caring for partners with heart disease expressed gratitude for still having each other and employed various coping strategies to navigate their new life situation. Maintaining a positive mindset and focusing on the positive aspects of life were highlighted as crucial for coping. There is a growing recognition of the positive aspects of caregiving, with caregivers reporting increased self‐esteem and personal growth from mastering caregiving tasks. Caregiving tasks may initially induce anxiety, but as caregivers become proficient in them, they can experience an elevation in their self‐esteem. Acquiring caregiving skills can also foster personal growth across different aspects of life. Despite the overwhelming nature of the caregiving role, many caregivers express a heightened sense of satisfaction when successfully handling their responsibilities. Moreover, the development of new skills has been found to fortify the bond between the caregiver and the person receiving care. Although spending a lot of time together can deepen the relationship, it can sometimes be isolating as well [7].

In summary, the study highlights the significant life changes experienced by spouses caring for partners with heart disease and adolescents at home. There seems to be a difference for spouses having children living at home, while at the same time managing serious parent disease. Older partner caregivers may have fewer conflicts related to work and children, allowing them to focus more on their own situation [30]. In contrast, younger spouses in this study faced the additional burden of simultaneously caring for adolescents and coping with their partner's illness, which presents a unique set of challenges. This suggests that younger families affected by serious illness may require specific types of support tailored to their needs.

## 6 Strength and Limitations of the Work

It is important to consider study limitations that could have influenced the results. Since we were not able to send original data in full between the countries, it limited our ability to study the original data thoroughly over time. Therefore, it was particularly important to meet on several occasions and have a close dialogue with the research team to reach a consensus and ensure confirmability [40].

The interviews were conducted in the participant's native language to ensure high quality and dependability [40]. There are quite a few differences between the Scandinavian languages but also many similarities, which enable us to understand each other well in the research team. Through the collaboration, a larger number of study participants could be included, which increases the transferability and credibility, and moreover, the authenticity [40]. Despite the difficulties of living in different countries with all that entails, we were able to overcome these challenges by engaging in open communication and collaboration throughout the analysis process.

## 7 Recommendations for Further Research

For further research, there are several promising avenues to explore. One possible focus could be to examine strategies to improve information sharing, provide emotional support and increase spousal involvement in patient care. Cross‐country comparisons of the experiences of young spouses could explore variations in healthcare systems, support structures and cultural influences. In addition, examining the impact of interventions such as counselling or support groups for young spouses of people with heart disease would provide valuable insights.

## 8 Implications for Policy and Practice

The study contributes to both policy and practice by offering insights into the Scandinavian context. Leveraging the region's tax‐financed healthcare systems and robust sick leave policies, policymakers can tailor support programs to better meet the needs of patients with heart disease and their families.

## 9 Conclusion

The findings shed light on the challenging life situation that spouses face when their partner has heart disease and adolescents living at home. Spouses became informal caregivers for their partner and this situation resulted in increased responsibilities in everyday life and a shift in the balance of parenthood. It is important to place this study in a broader context to fully understand its significance. The spouses expressed frustration and a sense of limitation in making plans, due to the unpredictable life situation. They also reported lacking support from healthcare professionals and a desire for guidance on how to involve the children. However, those who maintained a positive attitude and mindset towards life found it easier to cope with having a partner with heart disease and tried finding a new way of life.

## Author Contributions

**Matilda Holmbom:** methodology, validation, formal analysis, data curation, investigation, writing–original draft, visualisation. **Frida Andréasson:** methodology, validation, formal analysis, investigation, writing–review and editing, supervision. **Hanna Grundström:** methodology, supervision, formal analysis, writing–review and editing. **Camilla Bernild:** validation, formal analysis, investigation, writing–review and editing. **Nina Fålun:** validation, formal analysis, investigation, resources, writing–review and editing. **Tone Merete Norekvål:** validation, formal analysis, resources, writing–review and editing. **Selina Kikkenborg Berg:** conceptualisation, validation, formal analysis, resources, writing–review and editing, supervision, project administration, funding acquisition. **Anna Strömberg:** conceptualisation, methodology, validation, formal analysis, resources, writing–review and editing, supervision, project administration, funding acquisition.

## Conflicts of Interest

The authors declare no conflicts of interest.

## Data Availability

Data sharing is not applicable because of concerns about privacy and confidentiality related to qualitative interview data.
